# White Blood Cell-Based Detection of Asymptomatic Scrapie Infection by *Ex Vivo* Assays

**DOI:** 10.1371/journal.pone.0104287

**Published:** 2014-08-14

**Authors:** Sophie Halliez, Emilie Jaumain, Alvina Huor, Jean-Yves Douet, Séverine Lugan, Hervé Cassard, Caroline Lacroux, Vincent Béringue, Olivier Andréoletti, Didier Vilette

**Affiliations:** 1 INRA (Institut National de la Recherche Agronomique), UR892, Virologie Immunologie Moléculaires, Jouy-en-Josas, France; 2 UMR INRA ENVT 1225, Interactions Hôtes Agents Pathogènes, Ecole Nationale Vétérinaire de Toulouse, Toulouse, France; Rocky Mountain Laboratories, NIAID, NIH, United States of America

## Abstract

Prion transmission can occur by blood transfusion in human variant Creutzfeldt-Jakob disease and in experimental animal models, including sheep. Screening of blood and its derivatives for the presence of prions became therefore a major public health issue. As infectious titer in blood is reportedly low, highly sensitive and robust methods are required to detect prions in blood and blood derived products. The objectives of this study were to compare different methods - *in vitro*, *ex vivo* and *in vivo* assays - to detect prion infectivity in cells prepared from blood samples obtained from scrapie infected sheep at different time points of the disease. Protein misfolding cyclic amplification (PMCA) and bioassays in transgenic mice expressing the ovine prion protein were the most efficient methods to identify infected animals at any time of the disease (asymptomatic to terminally-ill stages). However scrapie cell and cerebellar organotypic slice culture assays designed to replicate ovine prions in culture also allowed detection of prion infectivity in blood cells from asymptomatic sheep. These findings confirm that white blood cells are appropriate targets for preclinical detection and introduce *ex vivo* tools to detect blood infectivity during the asymptomatic stage of the disease.

## Introduction

Transmissible spongiform encephalopathies (TSE) or prion diseases are deadly infectious neurodegenerative diseases naturally affecting a broad range of mammalian species including humans (Creutzfeldt-Jakob disease-CJD), goats, sheep (scrapie), cattle (bovine spongiform encephalopathy-BSE) and cervids (chronic wasting disease-CWD). Mammalian prions are thought to consist in aggregated and misfolded forms of a host protein, the prion protein (PrP), encoded by the *Prnp* gene. While TSE present sporadic and familial origins, they also can be acquired. Transmission can be achieved by several ways. In humans, a novel form of CJD, named variant (v) CJD and first reported in 1996, likely results from the infection by BSE agent through alimentation [1]. The subclinical period can vary from several weeks to decades depending of different parameters including the prion strain, the transmission route, the host species, the *Prnp* and possibly other polymorphisms. Importantly, prions replicate in tissues other than the nervous system, mainly lymphoid tissues. It is notably the case for vCJD and eventually the clinical phase could even not appear resulting in an asymptomatic carriage of vCJD infection [2], [3]. To date, there is no certitude about asymptomatic vCJD prevalence. Concerning the United Kingdom population where the reported number of BSE cases was the highest, recent data obtained from the survey of appendix tissue archives suggest a prevalence of subclinical infection in around 1 in 2000 in age-relevant population [4]. The detection of blood prions has been demonstrated in a number of experimentally or naturally infected animal species including rodents [5], [6], cervids [7], sheep [8], goats [9] and non-human primates [10]. Importantly, four cases of vCJD transmission were reported in individuals that were transfused with blood from asymptomatic donors that subsequently developed vCJD, strongly arguing that human blood can be infectious as well [11]–[13]. More recently, transgenic mice bioassay detected infectivity in some sporadic CJD plasma samples [14]. Even if the infectious titers in blood seem low (as assessed by intracerebral inoculation into susceptible mice), positive transmission following transfusion of as little as a few hundreds microliters of scrapie infected whole blood suggested that disease transmission by transfusion might be much more efficient than previously assumed [15]. Therefore prevention of vCJD transmission by blood transfusion is a major public health issue.

Distribution of prion infectivity among blood components may substantially vary depending on the host species and/or the prion agent. While previous studies in rodents [6], primates [10], cervids [16] and sheep [17] have clearly established that at least part of blood infectivity is associated with white blood cells (WBC), infectivity was detected in platelets from vCJD-infected mice [18], CWD-infected deer [16] and scrapie- [19] and BSE-infected sheep [20] but not in hamsters [21]. The contribution of plasma to blood infectivity also depends on the experimental paradigm. In rodents, up to fifty percent of blood infectivity is recovered from plasma [18] while plasma does not seem to be infectious in cervids [16]. In sheep, plasma is consistently infectious albeit less than whole blood [20]. For human, available data are scarce but suggest that plasma from CJD individuals may contain prions [14].

Sensitive and accurate detection of blood prions are required to design pertinent tests for non invasive early diagnostic, to improve the safety of blood transfusion and blood-derived products and to study the species/agent-dependant blood prionemia in infected animals or individuals. In the recent years, blood detection tests targeting abnormal PrP have been developed, some of which - Protein Misfolding Cyclic Amplification (PMCA) [22] and QuIC [23] - rely on prion-seeded amplification. Abnormal PrP has been PMCA-amplified from blood components of various asymptomatic animal species including rodents [24], sheep and cervids [25] while eQuIC allowed identification of positive plasmas from preclinical hamsters [26]. Abnormal PrP enrichment methods, such as plasminogen-based capture can be coupled to *in vitro* prion amplification to optimize the detection [27]. Of note, abnormal PrP was detected in vCJD blood samples by a steel binding/ELISA assay without any amplification step [28]. On the other hand, blood prions can also be detected by infectivity assays, notably for titration purposes. These assays involve blood transfusion in the original host [16], [29] or intracerebral inoculation of blood components into susceptible mice [6], [15], [16], [18], [29]. Quite recently, *ex vivo* assays have been developed to detect brain-derived prion infectivity without resorting to animal inoculation [30]–[32].

In this work, we aimed at comparing in the same experimental model the relative sensitivity and specificity of these different assays to detect prion in blood. We chose sheep experimentally infected with classical scrapie to isolate WBC and platelets at different stages of the incubation period. Infectivity of the different samples was tested by PMCA and bioassay in ovine PrP transgenic mice (tg338) mice. WBC were also subjected to the ovRK13 cell-based assay and to the Cerebellar Organotypic Slice Culture Assay (COSCA). Our data show that cell-based assay and COSCA are sensitive enough to detect prion infectivity in WBC from asymptomatic sheep. This study confirms the earlier finding that WBC are a suitable target for preclinical detection of infection and extends the number of complementary tools for prion detection in blood cells.

## Materials and Methods

### Ethics Statement

All animal experiments have been performed in compliance with our institutional and French national guidelines, in accordance with the European Community Council Directive 86/609/EEC. The experimental protocols were approved by the INRA Toulouse/ENVT ethics committees, and were performed in the approved animal facilities (C 31 555 27) of author's institution by an agreed experimentator (311055503). All efforts were made to minimize suffering.

### PG127 classical scrapie model

Oral inoculation of the PG127 sheep isolate has been described in detail previously [19]. Briefly, five 6- to 10-month-old TSE-free cheviot sheep were orally challenged with a 2-g equivalent of brain material (PG127 isolate previously endpoint titrated in tg338 mice). Animals were then observed until the occurrence of clinical signs (around 200 days post inoculation) and euthanized when exhibiting locomotor signs of the disease that impaired feeding. After culling, each sheep was necropsied and lymphoid and brain tissues were sampled for PrPSc detection.

### Blood collection, white blood cell and platelet preparation

Whole blood was collected from the jugular vein using citrate dextrose (35 mL) in a 250-mL blood collection pouch (MSE3500Q; Macopharma). Preparation of white blood cells and platelet samples were as described previously [19]. For WBC preparation, whole blood transferred into 15-ml conic tubes was centrifuged at 3,600 rpm for 10 min. Plasma was removed and the collected buffy coat was then was mixed with an equal volume of ACK solution (NH4CL, 0.15 M; KHCO3, 1 mM; Na2EDTA, 0.1 mM; pH 7.4) for 5 min at room temperature (RT). The obtained white blood cells then were washed 3 times with PBS. Cell aliquots were freezed at −80°C until used. For platelets, whole blood was centrifuged for 15 min at 300×g in 5-ml borosilicate tubes at room temperature. The top third of the plasma was collected and centrifuged for 30 min at 3,000×g. The supernatant was eliminated and the pellet resuspended in PBS (pH 7.4) before two additional washings. Pellets were resuspended in PBS, and the absence of both leukocytes and red cells was checked by direct microscopic examination in a Thomas' cell and Sysmex XT-2000i automat [19].

### Bioassay

Mouse bioassays were carried out in ovine VRQ PrP transgenic mice (tg338), which are highly sensitive for detection of PG127 sheep scrapie infectivity [15]. Six mice were intracerebrally inoculated with each sample (20 μL). Mice were clinically monitored until the occurrence of TSE clinical signs, at which time they were culled. Brain accumulation of PrP^res^ was confirmed in clinically-affected mice.

### PMCA

PMCA in a Misonix 4000 sonicator was carried out as previously described [19] with brain homogenate from tg338 mice as substrate. After the first round (96 cycles), the 2^nd^ round reactions were seeded with 1/10 of the first round. Samples were than processed for PrP^res^ analysis by immunoblotting.

### Organotypic Slice Cultures and COSCA

Cerebella were dissected from 10- to 12-day-old tg338 transgenic mice (tg338) over-expressing the VRQ allele of the ovine prion protein [33]. Preparation and culture of the slices were performed as described in the protocol published for the POSCA [30]. Briefly cerebella were embedded in 2.5%-low-melting-point agarose (Invitrogen) dissolved in Gey's balanced salt solution (Eurobio) supplemented with the glutamate receptor antagonist kynurenic acid (1 mM) (Sigma) and glucose (33 mM) (Sigma). 350-mm-thick slices were cut on a vibratome (HM650V, Microm), then recovered from the agarose and placed on 6-well Millicell culture inserts (Millipore) by groups of 8 to 10 slices. The inserts were transferred to a cell culture plate and cultured in sterile slice culture medium (SCM) composed of 50% minimum essential medium (Gibco), 25% basal medium Eagle (Gibco) and 25% horse serum (Gibco) and supplemented with glucose, penicillin/streptomycin and stable Glutamine (PAA). The slices were cultured at 37°C in a humidified atmosphere with 5% CO2 and the culture medium was exchanged three times a week.

Frozen pellets of WBC were resuspended in SCM at 5.10^5^ cells/μL. For quantification purposes, serial dilutions of brain stock prepared from terminally ill tg338 mice experimentally infected with PG127 scrapie strain and a 10^−2^ dilution of a brain from a non-infected tg338 mouse (negative control) were prepared in SCM. After 7 to 10 days in vitro (DIV), WBC suspensions (10^6^ cells/slice) and brain dilutions (2 μL/slice) were applied on slices (8 to 10 slices per condition). The slices were then culture for an additional 42 DIV.

Slices were harvested by scrapping and homogenized at 20 µg/μL in 5% sterile glucose solution with a Rybolyser (Hybaid, Middlesex, U.K.). For analysis, samples corresponding to 50μL of homogenate (∼2 slices equivalent) were processed then loaded on the gel. Briefly, PrP^Res^ was extracted by the Bio-Rad test protocol, by using 20 µg/mL proteinase K (PK) for 10 min at 37°C. After denaturation, the samples were run on 12% Criterion XT Bis-Tris gels (Bio-Rad), electrotransferred onto nitrocellulose membranes (Bio-Rad), and immunoblotted with biotinylated anti-PrP antibody Sha31b [34]. Immunoreactivity was revealed by chemiluminescence (reagents by Amersham Pharmacia Biosciences), imaged via a GeneGnome machine (Syngene) and the software GeneSnap (Syngene) and quantified via the software geneTools (Syngene).

### Cell-based assay

WBC (5×10^7^ cells) were homogenized in 200 μL of a 5% sterile glucose solution with a high-speed homogenizer (TeSeE Precess 48 system) and diluted to 800 μL with cell culture medium (Opti-MEM medium supplemented with 10% fetal bovine serum and 1 µg/mL of doxycycline). The cell-based assay procedure has been described previously [31]. Confluent ovRK13 cells grown in six-well plates were overlaid with 300 uL of the WBC homogenates. Two hours later, 300 μL of cell culture medium were added and the cells were incubated overnight. The inoculum was removed and the inoculation procedure was repeated once. After a further overnight incubation, 900 μL of cell culture medium were added and the cells were incubated for 5 more days. The inoculum was removed and fresh medium (3 mL with 1 µg/mL of doxycycline) was added. Infection was allowed to proceed for 3 more weeks with one medium change per week. Four weeks post inoculation, the cells from each well were trypsinized, cell pellets were resuspended in 500 μL of 5% sterile glucose and were homogenized with the high-speed homogenizer. Half of the resulting cell extracts (250 μL in 3 ml of culture medium with 1 µg/mL of doxycycline) was used to inoculate ovRK13 for a second round of cell assay. Four weeks later, cell cultures were rinsed with cold PBS and solubilized for 10 min at 4°C in Triton-DOC lysis buffer (50 mM Tris/HCl (pH 7.4), 0.5% Triton-X100, 0.5% sodium deoxycholate). The lysates were clarified by low speed centrifugation (425×*g*, 1 min) and cellular proteins in the post-nuclear supernatants were quantified by bicinchoninic acid (BCA, Pierce). Digestion of 750 µg of proteins with PK (recombinant grade, Roche) was performed for 2 h at 37°C with a mass ratio of 4 µg of PK per mg of cellular proteins and the reaction was stopped by addition of Pefabloc (Sigma-Aldrich) to 4 mM. PK-digested samples were centrifuged for 30 min at 20,000×*g* and pellets were analyzed by western blot. Samples separated by 12% SDS-PAGE electrophoresis were transferred to PVDF membranes (Bio-Rad). The western blots were stained for PrP with Sha31 mAb [34]. Filters were developed using an ECL+ reagent kit (Amersham-GE Healthcare) and visualized with a Bio-Rad VersaDoc imaging system.

## Results and Discussion

Five sheep, homozygous for the VRQ allele of ovine PrP, were orally challenged with 2 g of classical scrapie brain homogenate between 6 and 10 months of age. This brain homogenate (PG127 isolate), previously end-point titrated in tg338 mice, has a titer of 10^6.6^ ID_50_/g IC [35]. The five sheep (D1 to D5) were euthanized at terminal stage of prion disease at 198, 193, 198, 194 and 191 days post inoculation (dpi), respectively. Classical scrapie was confirmed by histopathology (vacuolar change in central nervous system) and detection of abnormal PrP deposit in the central nervous system and lymphoid tissues. At different time points during the asymptomatic phase (i.e. 50, 80, 130 days post inoculation (dpi)) and during the clinical phase of the disease (180 dpi), whole blood was collected from each recipient sheep and aliquots of platelets and WBC were prepared according to standard fractionation protocols. Four different methods were tested in parallel to detect PG127 prion infectivity or abnormal PrP in these samples: PMCA, mouse bioassay, Scrapie Cell Assay (SCA) and COSCA.

**Figure 1:**
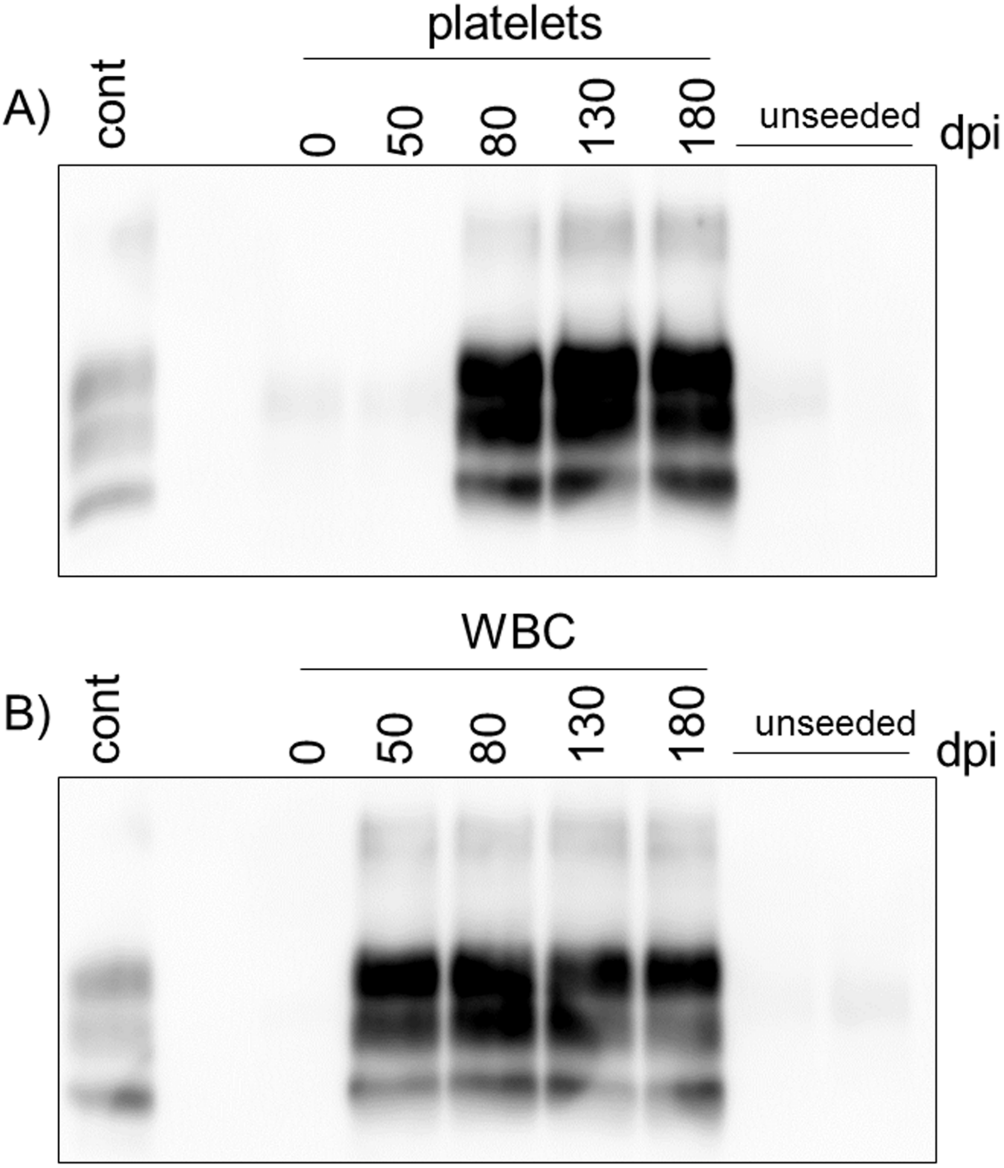
PMCA analysis of white blood cells and platelets samples. Platelets (A) and white blood cells (WBC) (B) from sheep D2 collected at the indicated time points (dpi) were subjected to two successive rounds of PMCA. Unseeded reactions were run in parallel. Samples were processed for PrP^res^ isolation and analyzed by immunoblotting. A western-blotting positive control (cont) is included in each gel.

Platelets samples were used to seed PMCA reactions using tg338 mice brain homogenate as substrate. Each sample, run in 5 replicates, was subjected to 2 successive rounds. The results are shown in table 1 with a typical immunoblot in fig 1A. PrP^res^ amplification was observed for all platelets samples collected 80 dpi onwards. Notably, consistent and robust detection was achieved even for platelets samples that showed incomplete transmission to tg338 mice (see below). This is a further illustration that PMCA is more sensitive than bioassay for detection of prions [36]–[38] including PG127 [39]. With 50 dpi samples, consistent PrP^res^ amplification was not achieved and no PrP^res^ was observed with platelets collected before inoculation. For the bioassay, groups of six tg338 transgenic mice over-expressing the VRQ allele of the ovine prion protein [33] were inoculated intracerebrally with each sample to be tested. Mice were monitored up to occurrence of TSE compatible clinical sign onset or killed 250 days post inoculation (i.e., approximately twice the incubation time at limiting dilution). All the mice were tested for presence of PK-resistant PrP (PrP^res^) deposition in brain. Platelet homogenates corresponding to 15 mL of plasma prepared at the different time points from the 5 donor sheep were inoculated and the results are presented in table 1. No transmission was observed with platelets collected before inoculation. All platelets samples collected at days 80, 130 and 180 transmitted the disease to the recipient mice, except for sheep D2 at 180 dpi. For most of the platelets samples the attack rate was not 100%. At 50 dpi, no transmission occurred with the platelets from 3 donor sheep and low transmission rate was observed with platelets from the 2 other sheep (D2 and D3).

**Table 1:** Evaluation of the infectivity present in platelets prepared from scrapie infected sheep by two different methods: PMCA and inoculation into tg338 mice (bioassay).

Donor		−1 dpi	50 dpi	80 dpi	130 dpi	180 dpi
**D1**	**PMCA**	0/5	0/5	5/5	5/5	5/5
	**Bioassay (tg338)**	0/6	0/6	6/6 (109±8)	5/6 (110±10)	1/6 (117)
**D2**	**PMCA**	0/5	1/5	5/5	5/5	5/5
	**Bioassay (tg338)**	0/6	1/6 (128)	6/6 (112±5)	1/6 (154)	0/6
**D3**	**PMCA**	0/5	3/5	5/5	5/5	5/5
	**Bioassay (tg338)**	0/6	2/6 (135,138)	4/6 (110±5)	6/6 (105±17)	4/6 (118±3)
**D4**	**PMCA**	0/5	0/5	5/5	5/5	5/5
	**Bioassay (tg338)**	0/6	0/6	5/6 (120±11)	6/6 (119±6)	1/6 (124)
**D5**	**PMCA**	0/5	0/5	5/5	5/5	5/5
	**Bioassay (tg338)**	0/6	0/6	1/6 (142)	3/6 (116±8)	2/6 (132,152)

Five susceptible VRQ/VRQ sheep were orally challenged with 2 g of brain homogenate (10^6.6^ ID_50_/g IC in tg*338* mice) between 6 and 10 months of age. The five VRQ/VRQ sheep respectively died at 198, 193, 198, 194 and 191days post inoculation (dpi). Classical scrapie was confirmed by histopathology (vacuolar change in central nervous system) and detection of abnormal PrP deposit in central nervous system and lymphoid tissues. At different time points, whole blood was collected from each donor and aliquots of platelets corresponding to 15 mL of plasma were prepared. Platelet homogenates (in 200μL) were inoculated in groups of six tg338 mice. Mice were monitored up to occurrence of TSE compatible clinical sign onset or killed at 250 days post inoculation. All mice were tested for presence of abnormal PrP deposition in brain. Incubation period in mice are presented in days (+/−SD). When less than 3 mice were positive, individual incubation period are given. In parallel the same homogenates were tested by PMCA (using tg338 mice brain homogenate as substrate). Each sample was run in 5 replicates for 2 successive rounds and the number of positive reactions is presented.

WBC homogenates were tested by PMCA in the same conditions than platelets. All WBC samples collected at 50 dpi and later lead to PrP^res^ amplification (table 2 and see fig 1B for a representative western blot). No PrP^res^ was detected when PMCA reactions were seeded with sheep WBC collected before experimental infection. These data demonstrate that PMCA allows a consistent and early detection of abnormal PrP in WBC from infected sheep. In bioassay experiments, infectivity was detected as early as 50 dpi in WBC of the 5 donor sheep, although the attack rate was not 100% for 3 of them (D1, D3 and D5) (table 2). At later time points, all WBC samples (except D2 at 180 dpi) transmitted the disease with 100% attack rates (table 2). However, mean incubation periods for D1, D3 and D4 samples at 180 dpi were statistically longer (p<0.05, Mann-Whitney-Wilcoxon test) than those of the corresponding 150 dpi samples, suggesting that WBC may be less infectious at 180 dpi. End-point titration of D5 WBC confirmed that WBC infectivity was close to the ID_50_ as the attack rates dropped after a single 10-fold dilution. Of note, only WBC collected at 130 dpi elicited transmission (1 out of 6) after 100-fold dilution. These results confirm the early and persistent prionemia associated with WBC already observed in this experimental model. In PMCA as in bioassay experiments, apparent infectivity associated with platelets is weaker than apparent infectivity associated with WBC, thus WBC only were used to compare the four different methods.

**Table 2:** Evaluation of the infectivity present in white blood cells prepared from scrapie infected sheep by four different methods: PMCA, SCA, Cerebellar Organotypic Slice Culture Assay (COSCA) and inoculation into tg338 mice (bioassay).

Donor			−1 dpi	50 dpi	80 dpi	130 dpi	180 dpi
**D1**	**PMCA**		0/5	5/5	5/5	5/5	2/5
	**SCA**		n.d.	Negative	Positive	n.d.	Positive
	**COSCA (tg338)**		n.d.	Negative	Positive	Positive	Positive
	**Bioassay (tg338)**		0/6	5/6 (106±9)	6/6 (93±4)	6/6 (95±2)	6/6 (101±2)
**D2**	**PMCA**		0/5	5/5	5/5	5/5	5/5
	**SCA**		n.d.	n.d.	Positive	Positive	n.d.
	**COSCA (tg338)**		n.d.	Negative	Positive	Positive	Negative
	**Bioassay (tg338)**		n.d.	6/6 (114±11)	6/6 (103±15)	6/6 (105±4)	5/6 (110±5)
**D3**	**PMCA**		0/5	5/5	5/5	5/5	5/5
	**SCA**		n.d.	n.d.	Positive	Positive	n.d.
	**COSCA (tg338)**		n.d.	Negative	Positive	Positive	Positive
	**Bioassay (tg338)**		n.d.	3/6 (122±6)	6/6 (100±7)	6/6 (88±7)	6/6 (103±12)
**D4**	**PMCA**		0/5	5/5	5/5	5/5	5/5
	**SCA**		n.d.	Negative	Positive	Positive	n.d.
	**COSCA (tg338)**		n.d.	Negative	Positive	Positive	Negative
	**Bioassay (tg338)**		n.d.	6/6 (104±8)	6/6 (85±2)	6/6 (99±2)	6/6 (111±4)
**D5**	**PMCA**		0/5	5/5	5/5	5/5	5/5
	**SCA**		n.d.	Negative	Negative	Positive	n.d.
	**COSCA (tg338)**		n.d.	Positive	Positive	Negative	Positive
	**Bioassay (tg338)**	neat	n.d.	2/6 (127,128)	5/5 (108±7)	6/6 (103±7)	6/6 (100±5)
		1/10	n.d.	0/6	3/6 (117±3)	5/6 (113±8)	4/6 (129±9)
		1/100	n.d.	0/6	0/6	1/6 (125)	0/6
		1/1000	n.d.	0/6	0/6	0/6	0/6

Five susceptible VRQ/VRQ sheep were orally challenged with 2 g of brain homogenate (10^6.6^ ID_50_/g IC in tg*338* mice) between 6 and 10 months of age. The five VRQ/VRQ sheep respectively died at 198, 193, 198, 194 and 191days post inoculation (dpi). Classical scrapie was confirmed by histopathology (vacuolar change in central nervous system) and detection of abnormal PrP deposit in central nervous system and lymphoid tissues. At different time points whole blood was collected from each donor and aliquots of 10^8^ white blood cells corresponding to 15 mL of plasma were prepared. These cells were tested by *in vitro* (PMCA), *ex vivo* (SCA and COSCA) and *in vivo* (bioassay) approaches in parallel. For PMCA (using tg338 mice brain homogenate as substrate), each sample was run in 5 replicates for 2 successive rounds and the number of positive reactions is presented. For SCA, 4×10^7^ white blood cells were inoculated to ovRK13 cells and abnormal PrP accumulation was assayed 60 days later. For COSCA, 8–10 cerebellar slices prepared from tg338 pups received 10^6^ cells (in 2μL) each and were cultured for 42 days then tested for the presence of abnormal PrP. For bioassay, 10^8^ white blood cells (in 200μL) were inoculated in groups of six tg338 mice. Mice were monitored up to occurrence of TSE compatible clinical sign onset or killed after 250 days post inoculation. All mice were tested for presence of abnormal PrP deposition in brain. Incubation period in mice are presented in days (+/−SD). When less than 3 mice were positive, individual incubation periods are given. Not done: n.d.

**Figure 2:**
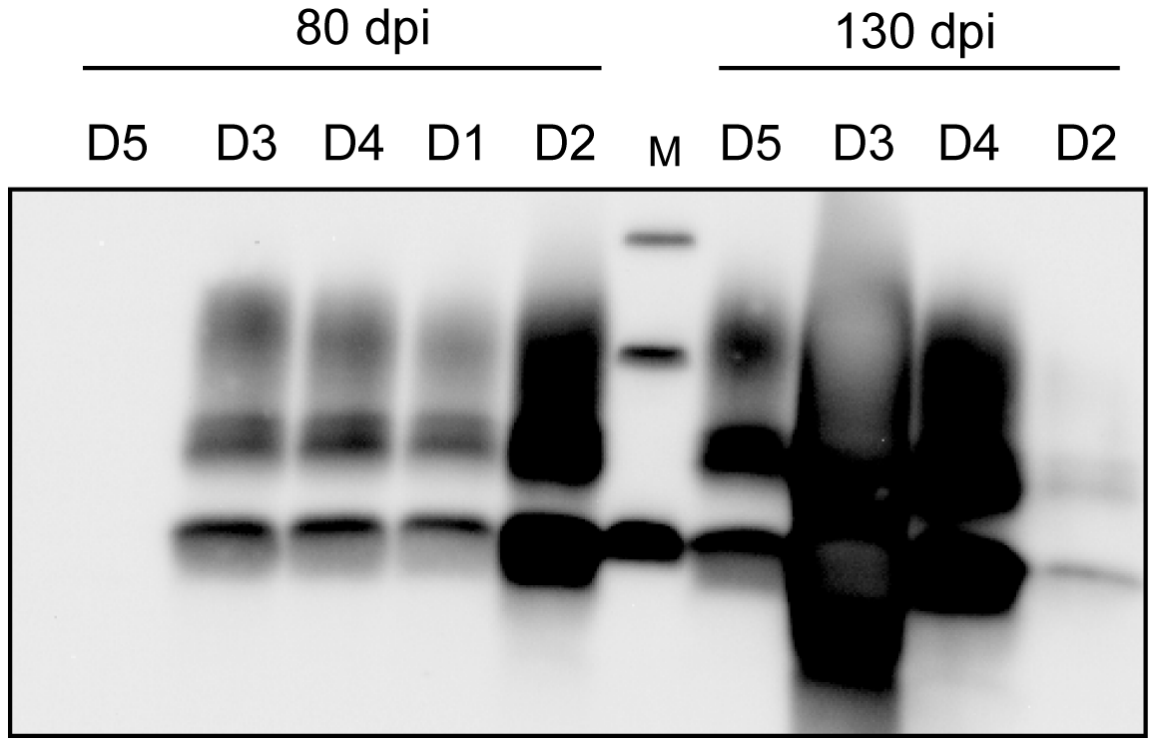
Cell-based assay of white blood cells infectivity from asymptomatic scrapie sheep. White blood cells from 5 infected sheep (D1 to D5) were isolated 80 days and 130 days post inoculation (dpi) when sheep were still asymptomatic. White blood cell homogenates (4×10^7^ cells) were inoculated to recipient ovRK13 cells. After 2 successive rounds of cell assay, the cultures were assayed for PrP^res^ by immunoblotting. PrP^res^ level is higher in cells infected with D3 130 dpi sample but its banding pattern is similar to that in cells infected with the other samples. M are molecular mass marker proteins (20, 30 and 40 kDa).

To determine if WBC infectivity could also be detected through a cell-based assay, we used a cell-based procedure recently developed for sensitive detection of PG127 prions [31]. In this paradigm, recipient ovRK13 cells were inoculated with WBC homogenates collected at different time points as for tg338 mouse bioassay (table 2). Inoculated ovRK13 cells were maintained for 8 weeks before being solubilized and assayed for the presence of PrP^res^ as a marker of prion infection. The large majority (9 out of 10) of the WBC samples assayed and collected at days 80, 130 and at the terminal stage of the disease (180 dpi) lead to infection of the recipient ovRK13 cells, as assessed by western blot detection of PrP^res^ (see fig. 2 for a representative immunodetection). Three samples collected at 50 dpi were also tested in parallel and did not lead to detectable PrP^res^ in the inoculated ovRK13 cells (table 2).

**Figure 3:**
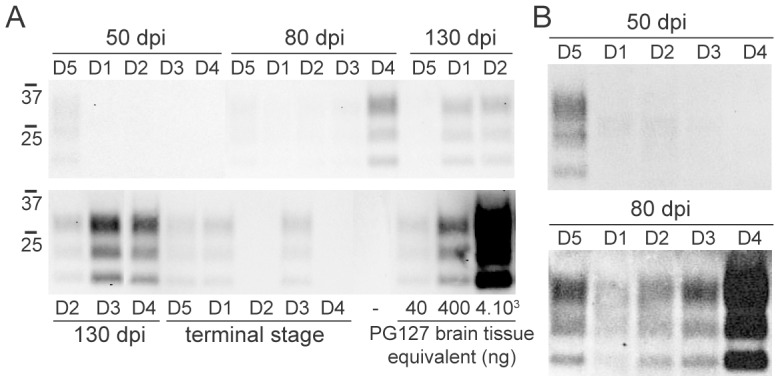
Evaluation of the infectivity present in white blood cells prepared from scrapie infected sheep by Cerebellar Organotypic Slice Culture Assay. Immunoblots of PK-treated slice culture homogenates probed with anti-PrP antibody Sha31, showing PrP^res^ accumulation in slice culture. (A) Cerebellar organotypic slices were prepared from tg338 pups and maintained in culture during 42 days *in vitro* after exposure to white blood cells prepared from blood collected from five scrapie infected sheep (D1, D2, D3, D4 and D5) at different times: 50 days post inoculation (dpi), 80 dpi, 130 dpi and at the terminal stage (180 dpi). For quantification purposes, slice cultures were also exposed to serial dilutions of PG127 scrapie-infected brain stock prepared from terminally ill tg338 mice, previously used [31]. To visualize low levels of PrP^res^, membranes were exposed over-night (B).

We adapted COSCA from the Prion Organotypic Slice Culture Assay (POSCA) [30]–[32], an *ex vivo* method allowing to titrate prions in a cerebral tissue context. Prion infection with WBC or brain homogenate was performed 7 to 10 days after cerebellar organotypic slices were established from tg338 mice. Then the slices were cultured for 42 additional days in vitro (DIV) and the presence of PrP^res^ was assessed by western blot analysis. We first compared, by endpoint titration the sensitivity of the tg338-derived COSCA to that of the tg338 mouse bioassay, by using a pool of 127S brain homogenate (the strain derived from PG127 serial transmission to tg338 mice and cloned by limiting dilution). The equivalent of 4 nanograms of 127S-infected tg338 brain was sufficient to elicit PrP^res^ detection in slice cultures after 42 DIV (Fig. S1, n = 2). By comparison, the limiting dilution for tg338 mice transmission corresponds to 2 ng of brain tissue [31]. This confirmed the high sensitivity of the COSCA assay to titrate prions. WBC extracts, collected at different time points from the five donor sheep, were directly loaded onto the slices along with serially diluted sheep PG127 scrapie-infected brain homogenate. As observed with the SCA, a high proportion (12 out of 15) of the samples collected between 80 dpi and the end stage (180 dpi) elicited detectable PrP^res^ accumulation in slice cultures (table 2). Only one (D5) out of the five samples collected at 50 dpi was positive by COSCA (fig. 3A, B). Slices infected with D2, D3 and D4 WBC collected at 130 dpi accumulated higher PrP^res^ levels, consistent with the higher infectivity detected by bioassay. PrP^res^ levels in slices infected with 130 dpi samples were equivalent to those observed after infection with 40 to 400 ng of PG127 brain tissue equivalent, while 80 dpi and end stage samples were less positive than those infected with 40 ng of PG127 brain tissue equivalent (fig. 3A,B).

All of the methods tested in this work confirm that WBC are the most suitable candidates to screen for blood prion infectivity during the asymptomatic stage of the disease. Among the different methods, PMCA (2 rounds) and mouse bioassay are the most sensitive as they consistently detect abnormal PrP and infectivity, respectively, at the first time point (50 dpi) tested in this study. Our results demonstrate that *ex vivo* methods (cell-based assay and COSCA) are sensitive enough to detect prion infectivity in WBC of preclinical scrapie sheep, and as such represent an ethical alternative to mouse bioassay to study blood prionemy. Studies with cell culture models have reported that live cells transmit infection much more efficiently than dead cells [40], [41]. Further experiments are required to determine whether sensitivity of cell-based and COSCA assays may be improved by using freshly-isolated instead of frozen pellets of WBC.

## Supporting Information

Figure S1 **Sensitivity of the Cerebellar Organotypic Slice Culture Assay.** Immunoblots of PK-treated slice culture homogenates probed with anti-PrP antibody Sha31, showing PrP^res^ accumulation in slice cultures. Cerebellar organotypic slices were prepared from tg338 pups and maintained in culture during 42 days *in vitro* after exposure to serial dilutions of PG127 scrapie-infected brain stock prepared from terminally ill tg338 mice, previously used [31].
(TIF)
