# Supplementary figures and images for: White Blood Cell-Based Detection of Asymptomatic Scrapie Infection by Ex Vivo Assays
Source: PLoS One. 2014 Aug 14;9(8):e104287. doi: 10.1371/journal.pone.0104287 (PMC4133197; doi:10.1371/journal.pone.0104287)

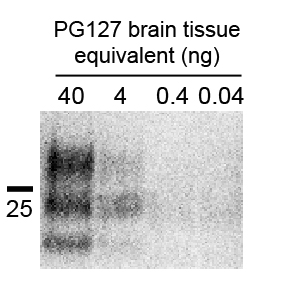

Supplement: Figure S1 — Sensitivity of the Cerebellar Organotypic Slice Culture Assay. Immunoblots of PK-treated slice culture homogenates probed with anti-PrP antibody Sha31, showing PrPres accumulation in slice cultures. Cerebellar organotypic slices were prepared from tg338 pups and maintained in culture during 42 days in vitro after exposure to serial dilutions of PG127 scrapie-infected brain stock prepared from terminally ill tg338 mice, previously used [31]. (TIF) [file pone.0104287.s001.tif]
